# A Comparison of Intraoperative 3D and Conventional 2D Fluoroscopy to Detect Screw Misplacement in Volar Plate Osteosynthesis of the Distal Radius

**DOI:** 10.3390/jcm14165896

**Published:** 2025-08-21

**Authors:** Fenna Brunken, Benno Bullert, Livia Morlock, Jula Gierse, Paul A. Grützner, Sven Y. Vetter, Nils Beisemann

**Affiliations:** BG Klinik Ludwigshafen, Department for Orthopaedics and Trauma Surgery at Heidelberg University, Ludwig-Guttmann-Str. 13, 67071 Ludwigshafen, Germany; fenna.brunken@bgu-ludwigshafen.de (F.B.); benno.bullert@bgu-ludwigshafen.de (B.B.); livia.morlock@bgu-ludwigshafen.de (L.M.); jula.gierse@bgu-ludwigshafen.de (J.G.); paul.gruetzner@bgu-ludwigshafen.de (P.A.G.); sven.vetter@bgu-ludwigshafen.de (S.Y.V.)

**Keywords:** distal radius fractures, intra-operative imaging, screw misplacement, 3D fluoroscopy, CBCT

## Abstract

**Background/Objectives**: Dorsal screw protrusion or intra-articular screw penetration at the distal radius can cause extensor tendon injuries or articular surface damage. Despite the use of various views, the detection of screw misplacement remains limited in 2D fluoroscopy. This study compares the sensitivity of 2D and 3D fluoroscopy for detecting screw misplacement at the distal radius. **Methods**: Volar locking plates were placed in six cadaveric forearms, and dorsal or intra-articular screw misplacement was induced. For each screw position, images were acquired by 2D and 3D fluoroscopy and assessed by three blinded observers. Sensitivity and specificity, inter-rater agreement, and observer confidence were evaluated. The dose area product (DAP) was measured separately for 2D and 3D fluoroscopy. **Results**: Three-dimensional fluoroscopy showed higher sensitivities for detecting dorsal (97.22%) and intra-articular (95.83%) screw misplacements than two-dimensional fluoroscopy. In 2D fluoroscopy, sensitivity for detecting dorsal screw protrusions improved from 63.89 to 75.00–77.78% with the inclusion of tangential views. For intra-articular penetrations, sensitivity in 2D fluoroscopy increased from 79.17 to 83.33% with the addition of oblique views. Observer confidence was higher in 3D fluoroscopy. DAP was significantly higher in 3D (42.4 ± 0.4 cGycm^2^) compared to 2D fluoroscopy (14.2 ± 3.7 cGycm^2^) (*p* < 0.0001). **Conclusions**: Compared to 2D fluoroscopy, 3D fluoroscopy improves the detection of screw misplacement at the distal radius. However, its routine use is constrained by increased radiation exposure and limited availability. If 3D fluoroscopy is not accessible, the addition of dorsal tangential and oblique views may improve the sensitivity of 2D fluoroscopy.

## 1. Introduction

Distal radius fractures represent the most common type of fracture in adults and frequently require surgical intervention [1,2]. Open reduction and internal fixation with volar locking plates has become the most common procedure in the surgical management of distal radius fractures [3,4]. Despite the advantages of volar plate osteosynthesis, it is associated with potential complications, including screw misplacement, possibly resulting in tendon injuries or destruction of the articular surface [5].

Previous studies have shown perforations of the dorsal cortex in 9 to 37% of screws placed despite using a depth gauge [6,7,8]. Dorsal screw prominence can lead to irritation and ruptures of extensor tendons, particularly the extensor pollicis longus (EPL) tendon. The incidence of EPL tendon ruptures reported in the literature ranges from 0.38 to 12.5% [9,10,11,12].

Detection of screw protrusion into the third extensor compartment can be challenging in standard lateral fluoroscopic views, since the EPL groove may be obscured by the Lister’s tubercle [13]. To improve the detection of dorsal screw prominence, the use of additional fluoroscopic views like the dorsal tangential view (DTV), carpal shoot through view (CSV), radial groove view, and oblique views has been proposed [6,14,15,16].

In addition to the risk of dorsal screw prominence, there is a risk of intra-articular screw perforation into the radiocarpal joint, which can result in cartilage damage and subsequent osteoarthritis [8]. Due to the biconcave articular surface of the distal radius, intra-articular screw perforations can be difficult to assess.

Although previous studies have shown that additional fluoroscopic views may improve the detection of screw misplacement, the reliability of conventional intraoperative fluoroscopy in identifying misplaced screws remains limited [8,17]. Intraoperative three-dimensional (3D) fluoroscopy has gained popularity for the assessment of fracture reduction and implant positioning, especially in complex fractures. Previous studies have demonstrated that intraoperative 3D imaging may reveal misplaced screws that were not detected in 2D fluoroscopy, prompting intraoperative screw revisions [18,19]. However, the use of intraoperative 3D fluoroscopy is limited by its availability and investment costs, and previous studies have shown that the operating time was interrupted by an average of three minutes for performing and analyzing the intraoperative 3D scan [19]. Still, the optimal intraoperative imaging protocol to detect screw misplacement remains controversial [16].

The aim of this study was to compare the accuracy of detecting dorsal and intra-articular screw misplacement in a cadaveric forearm model using different 2D fluoroscopic views and 3D fluoroscopy with a C-arm cone beam computed tomography (CBCT). To reflect clinical practice more realistically, conventional fluoroscopy images were analyzed as single images and as combinations of multiple views. Additionally, observer confidence and inter-rater reliability were assessed.

## 2. Materials and Methods

### 2.1. Specimens

Six fresh-frozen human forearm specimens were obtained from three body donors. This study was approved by the responsible ethics committee (application number 2024-17547). The upper extremities were disarticulated at the glenohumeral joint, and the specimens were thawed at room temperature for 24 h prior to use. Radiologic evaluation showed a distal ulna fracture in one specimen. No pre-existing fractures or bone abnormalities were observed in the distal radius of any specimen.

### 2.2. Preparation

Variable-angle locking plates (2.4 mm VA-LCP Two-Column Volar Distal Radius Plates (DePuy Synthes, Raynham, MA, USA)) were placed on the distal radius of all six forearms using a flexor carpi radialis approach. The plates are pre-contoured to match the volar surface of the distal radius and were positioned just proximal to the watershed line. The distal screw holes allow variable-angle locking screw placement to provide subchondral support of the distal articular surface. All surgical procedures were performed by an experienced trauma surgeon. To allow dorsal screw protrusion, the distal locking screw holes were drilled bicortically using a variable-angle drill sleeve, and locking screws were placed in the four most distal plate holes. Screw length was initially determined using a depth gauge, and implant position was verified using standard 2D fluoroscopy. Screw positioning was confirmed by direct visualization of the dorsal cortex and articular surface via a dorsal approach. If misplaced screws were identified, screw lengths were adjusted until no protrusion was evident.

### 2.3. Dorsal Protrusion

**Figure 1:**
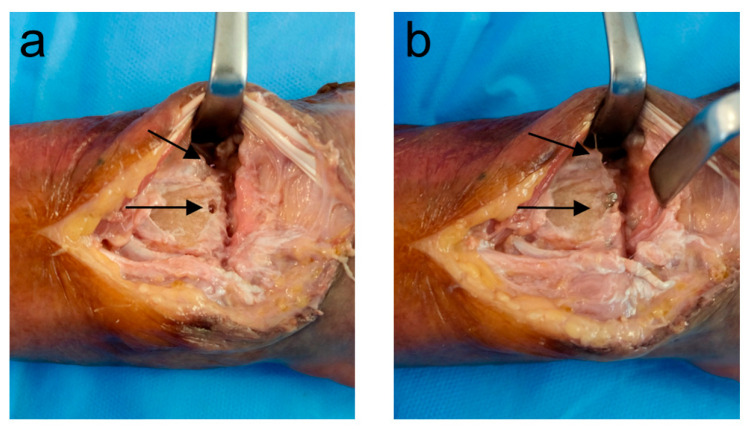
Visualization of the dorsal cortex shows a protrusion of 2 screws. (**a**) Grade B protrusion (1–2 mm); (**b**) Grade C protrusion (>2 mm).

Initial screw placements without dorsal protrusion were classified as Group A. Standard 2D fluoroscopic views and a 3D scan were acquired as described below. Subsequently, two screws per specimen were replaced with screws that were 2 mm longer to create a dorsal protrusion of 1–2 mm (Group B). Using a randomization table, one radial screw (screw 1 or 2) and one ulnar screw (screw 3 or 4) were randomly selected to create a dorsal protrusion in each specimen. Screw protrusion was confirmed by direct visualization, as displayed in Figure 1. After imaging, the same screws were exchanged for screws that were 1 mm longer than those in Group B to achieve a dorsal protrusion of >2 mm (Group C). The same imaging protocol was used for each screw position.

### 2.4. Intra-Articular Penetration

To induce intra-articular screw misplacement, the screw length and trajectory were planned to achieve a perforation of the articular surface of 1–3 mm based on the 3D scans. In each specimen, two screws were exchanged consecutively, perforating the lunate and scaphoid fossa, respectively. Then, 2D and 3D imaging were performed for each screw position, as described below.

### 2.5. Imaging

**Figure 2:**
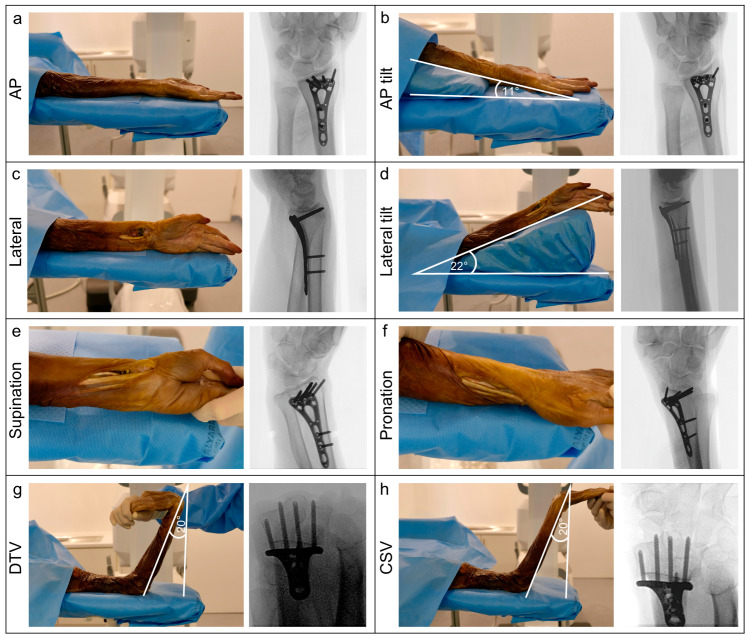
Display of the different 2D fluoroscopic views. (**a**) AP view; (**b**) Tilted AP view; (**c**) Lateral view; (**d**) Tilted lateral view; (**e**) Supinated view; (**f**) Pronated view; (**g**) Dorsal tangential view (DTV); (**h**) Carpal shoot through view (CSV).

**Figure 3:**
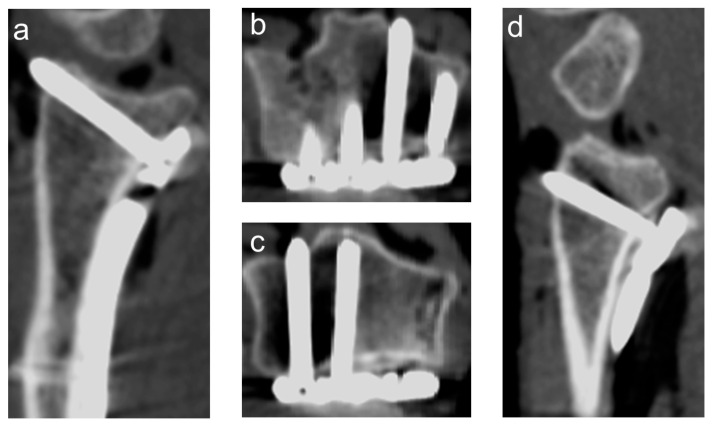
Example of a 3D scan with a protrusion of the second and fourth screw (counting from radial). (**a**) sagittal view screw of the second screw; (**b**) axial view of the second screw; (**c**) axial view of the fourth screw; (**d**) sagittal view of the fourth screw.

**Figure 4:**
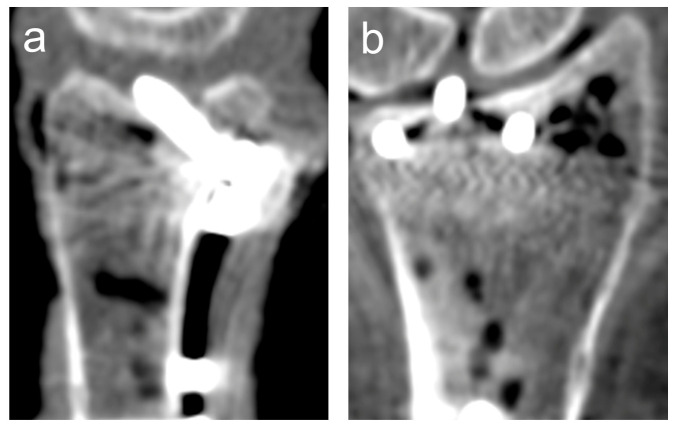
Example of a 3D scan showing an intra-articular penetration of the third screw (counting from radial). (**a**) sagittal view; (**b**) coronal view.

A 3D scan was acquired using a mobile 3D C-arm CBCT (Cios Spin, Siemens Healthineers, Erlangen, Germany). For each specimen, eight standardized fluoroscopic views were acquired: anteroposterior (AP), AP tilt, lateral, lateral tilt, oblique (pronation and supination), dorsal tangential (DTV), and carpal shoot-through views (CSV). Then, 2D fluoroscopy was performed by the same surgeon in all specimens, and the position of the C-arm and wrist was adjusted until optimal image quality was achieved. Foam positioning aids were used to achieve the correct angulation for tilted views. The positions for the different fluoroscopic views are shown in Figure 2. Examples of 3D scans showing dorsal screw protrusion or intra-articular screw misplacement are shown in Figure 3 and Figure 4.

### 2.6. Evaluation

The images were evaluated by three independent observers (two trauma surgery residents and one consultant) blinded to screw positioning. All data sets were anonymized and randomly sorted.

### 2.7. Dorsal Protrusion

For each screw position, 3D scans, single fluoroscopic images, and three different combinations of fluoroscopic views were presented. Set 1 consisted of AP, lateral and oblique (pronation/supination) views; Set 2 combined AP, lateral, DTV, and CSV images; Set 3 included all views (AP, lateral, oblique (pronation/supination), DTV, and CSV). In total, 18 3D scans, 144 single fluoroscopy images, and 54 fluoroscopic image sets were evaluated by each observer for dorsal protrusion.

### 2.8. Intra-Articular Penetration

For the evaluation of intra-articular screw penetration, each observer assessed 22 3D scans, 132 single 2D fluoroscopy images (AP, AP tilt, lateral, lateral tilt, pronation, and supination), and 38 fluoroscopic image sets. Set 1 consisted of AP and lateral views, and Set 2 combined AP, lateral, and oblique views (pronation/supination).

For the 3D scans, multiplanar reconstructions were displayed, and observers were allowed to adjust the reconstruction planes at their discretion. To measure screw penetrations in 3D scans, the reconstruction plane was aligned parallel to the penetrating screw. In axial views, a line was constructed along the dorsal cortex and a second parallel line at the tip of the screw, following the method described by Langerhuizen et al. [20]. The distance between the lines was measured in mm.

Observers were asked to answer the following questions: (1) Is there a penetration of the dorsal cortex? (2) Is there a penetration of the distal radial articular surface? The confidence in the assessment of screw positioning was rated on a 5-point Likert scale by each observer (criteria shown in Table 1). The observers were instructed to measure the screw protrusion in 3D scans. The dose area product (DAP) from the dose reports was analyzed separately for 2D and 3D fluoroscopy.

### 2.9. Statistics

Data were analyzed using Prism 10 (GraphPad, San Diego, CA, USA) and R 4.3.2 (R Core Team, Vienna, Austria). The normal distribution was tested using the Shapiro–Wilk Test. Descriptive statistics are shown as means ± standard deviation for normally distributed data or median with interquartile range (IQR) for non-normally distributed data. Multiple comparisons of non-normally distributed data were analyzed using a Friedman test with Dunn’s post hoc correction. A paired *t*-test was used to compare normally distributed data between two groups.

To evaluate factors affecting the detection of intra-articular and dorsal screw protrusion, two separate logistic regression models with robust standard errors clustered by individual screw misplacement models were used. Fixed effects included imaging modality (2D/3D), rater, and the degree of dorsal screw protrusion (1–2 mm, >2 mm) or location of intra-articular protrusion (lunate/scaphoid fossa), respectively. Based on the observed effect sizes, a post hoc power analysis was conducted.

Inter-rater reliability was assessed using Fleiss’ kappa for nominal data and the Intraclass Correlation Coefficient (ICC) for continuous data. ICC values were calculated using a two-way mixed effects model for absolute agreement of single measurements. Fleiss’ kappa was interpreted following Landis and Koch [21], and ICC was interpreted according to Cicchetti’s recommendations [22]. *p*-values < 0.05 were considered statistically significant.

## 3. Results

### 3.1. Dorsal Protrusion

#### 3.1.1. Sensitivity and Specificity

The overall sensitivity and specificity for detecting dorsal screw protrusion were higher in 3D fluoroscopy compared to all combinations of conventional fluoroscopic views. Sensitivity was also higher for protrusions >2 mm than for 1–2 mm, particularly in 2D fluoroscopy. The inclusion of DTV and CSV improved the sensitivity of 2D fluoroscopy. The detailed results are provided in Table 2. Logistic regression showed significant effects of imaging modality (3D/2D) (all *p* < 0.01, β = 2.84–3.51) and degree of protrusion (*p* < 0.001, β = 3.31) on the detection of dorsal screw protrusion. A post hoc power analysis based on the observed effect sizes indicated high power (> 0.88) for detecting differences in the detection of dorsal screw protrusion.

#### 3.1.2. Inter-Rater Agreement

Inter-rater agreement for the detection of dorsal screw protrusion was almost perfect in 3D fluoroscopy (Fleiss’ Kappa: 0.92) and substantial for 2D fluoroscopy (Fleiss’ Kappa: 0.62–0.70). The ICC for measurement of protrusion length in 3D scans was excellent (ICC = 0.89, 95% CI: 0.84–0.92).

#### 3.1.3. Observer Confidence

There was a significant difference in observer confidence for the detection of dorsal screw protrusion (Friedman test; Chi2 = 45.97, Kendall’s W = 0.28, *p* < 0.0001). Post hoc pairwise comparisons using Dunn’s test showed that observer confidence was significantly higher in 3D scans compared to Set 1 (r = 0.75, *p* < 0.0001) and Set 2 (r = 0.50, *p* = 0.0016). In 2D fluoroscopy, observer confidence was higher for Set 3 compared to Set 1 (r = 0.51, *p* = 0.0012).

#### 3.1.4. Comparison 2D Fluoroscopic Views

The analysis of single 2D fluoroscopic views showed a sensitivity of 100% for DTV. Although the overall sensitivity for CSV was lower (69.44%), it also showed a sensitivity of 100% for protrusions > 2 mm. In oblique views, the sensitivity was 97.22% for supinated compared to 33.33% for pronated views. Specificity ranged from 61.11% to 94.44%.

Inter-rater reliability varied depending on the view, ranging from slight (pronation, κ = 0.08) to almost perfect (CSV, κ = 0.93).

A significant difference in observer confidence was found across views (Friedman test; Chi2 = 72.87, Kendall’s W = 0.27, *p* < 0.0001). Post hoc pairwise comparisons (Dunn’s test) showed that observer confidence was higher for DTV, CSV, and supinated views compared to pronated and lateral/lateral tilt views (all *p* < 0.05, r = 0.18–0.78). The results are presented in Table 3. 

### 3.2. Intra-Articular Penetration

#### 3.2.1. Sensitivity and Specificity

Sensitivity and specificity for detecting intra-articular screw penetration were higher in 3D fluoroscopy compared to 2D fluoroscopy. The addition of pronated and supinated views increased the sensitivity and specificity of 2D fluoroscopy. Detailed results are shown in Table 4. Logistic regression showed a smaller effect of image modality on the detection of intra-articular screw misplacement compared to dorsal screw protrusion (Set 1: *p* = 0.078, β = −1.43; Set 2: *p* = 0.07, β = −1.36). There was no significant effect of screw location on detection (*p* = 0.515, β = 0.77). A post hoc power analysis indicated lower power (0.66) for detecting differences between imaging modalities for the detection of intra-articular screw misplacement.

#### 3.2.2. Inter-Rater Agreement

Fleiss’ Kappa showed a substantial to almost perfect inter-rater agreement for both 2D and 3D fluoroscopy (0.71–0.86). The ICC for measurements of intra-articular screw penetration in 3D scans was excellent (0.94, 95% CI: 0.89–0.97).

#### 3.2.3. Observer Confidence

A significant difference in observer confidence for detecting intra-articular screws was found (Friedman test; Chi2 = 22.19, Kendall’s W = 0.42, *p* < 0.001). Confidence was significantly higher in 3D fluoroscopy compared to both 2D sets (r = 0.36, *p* = 0.0073). There was no significant difference between Set 1 and Set 2 (r = 0.0, *p* > 0.99).

#### 3.2.4. Comparison 2D Fluoroscopic Views

For the detection of intra-articular screws in single fluoroscopy views, the highest sensitivity (84.85%) and specificity (100%) were found for pronated views. Tilted AP and lateral views showed a higher sensitivity and specificity compared to conventional AP and lateral views. The results are presented in Table 5. Comparison by screw perforation location showed that pronated views achieved a sensitivity of 93.33% for detecting screws penetrating the lunate fossa compared to 77.78% for screws in the scaphoid fossa, whereas supinated views showed a sensitivity of 66.67% for perforations in the lunate fossa and 77.78% for the scaphoid fossa.

Inter-rater agreement ranged from fair (lateral tilt: κ = 0.33) to almost perfect (AP: κ = 0.87). Observer confidence differed significantly across the six views (Friedman test; Chi2 = 15.98, Kendall’s W = 0.10, *p* = 0.0069). A lower confidence was observed in lateral and lateral tilt views. However, post hoc pairwise comparisons did not show any statistically significant differences between the views.

### 3.3. Radiation Exposure

The DAP was significantly higher for intraoperative 3D scans compared to 2D fluoroscopy (mean 3D: 42.37 ± 0.37 cGycm^2^, mean 2D: 14.16 ± 3.72 cGycm^2^, unpaired *t*-test, Cohen’s d = 10.67, *p* < 0.0001).

## 4. Discussion

Volar locking plates are routinely used for the surgical treatment of distal radius fractures. Still, complications such as dorsal screw protrusion and intra-articular screw misplacement may adversely affect clinical outcomes. Previous studies have reported dorsal screw penetration in up to 37% and intra-articular screw misplacement in 14% of cases after volar plate osteosynthesis of intra-articular distal radius fractures [8].

Dorsal screw prominence can cause irritation and subsequent ruptures of extensor tendons, particularly the extensor pollicis longus (EPL) tendon, which usually requires revision surgery [23].

Intra-articular screw penetration occurs more frequently in complex distal radius fractures and correlates with the degree of osteoarthritis [8,17]. To achieve optimal biomechanical stability, distal locking screws are placed close to the joint surface, increasing the risk of intra-articular perforation and subsequent cartilage damage [24].

To prevent these complications, fluoroscopy is used intraoperatively to assess implant positioning to allow for immediate revision. However, previous studies have demonstrated the limitations of standard anteroposterior and lateral fluoroscopic images in detecting screw misplacement in the distal radius [16,20,25]. The current study adds to previous data by comparing the detection of intra-articular and dorsal screw protrusion among eight different conventional fluoroscopic views and between commonly used combinations of 2D fluoroscopy views and 3D fluoroscopy under standardized conditions while also assessing observer confidence and inter-rater reliability.

### 4.1. Detection of Dorsal Screw Protrusion in 2D Fluoroscopy

Due to superimposition by Lister’s tubercle, an average screw protrusion of 3.5 mm is needed in the EPL groove to be detected in lateral views [26]. The high anatomic variability of Lister’s tubercle and dorsal fracture comminution can complicate the evaluation of the dorsal cortex [9,13,27].

The current study showed sensitivities of 58.3% for standard lateral and 52.78% for tilted lateral views, comparable to the results of Hill et al. (58.7%), while other studies reported lower sensitivities of only 18.2–24.5 % [7,28,29].

In this study, supinated views showed higher sensitivity (97.22%) compared to pronated views (33.33%), aligning with previous studies demonstrating higher sensitivities for supinated (40–88.2%) than pronated (12–53.4%) views in detecting dorsal screw protrusion [29,30,31]. Oblique views in supination and pronation are used to improve the detection of screw protrusion into the second and fourth compartments, respectively [30,32].

Supinated views are particularly useful for evaluating the radial dorsal cortex and the position of radial styloid screws due to reduced superimposition of the dorsal cortex. However, this does not account for the observed difference in sensitivity of pronated and supinated views in the current study, as the locations of protruding screws were distributed equally.

Screws penetrating the third compartment can still be missed using oblique views; therefore, DTV has been recommended for detecting dorsal protrusions [16,32,33,34]. DTV is acquired with the fluoroscopy beam aimed tangentially at the distal radius, enabling evaluation of the third extensor compartment without superimposition of Lister’s tubercle.

In this study, DTV showed the highest sensitivity for detecting dorsal screw protrusion (100%), while CSV had a lower overall sensitivity (69.44%). Previous studies have reported sensitivities ranging from 66.5 to 100% for DTV [6,7,20,29,32,35,36,37].

Clinical studies demonstrated that adding DTV to standard fluoroscopic views prompted an exchange of protruding screws in 15–27% of cases and reduced residual screw protrusions in postoperative CT scans [14,20,25,34,38].

In contrast to our results, Stoops et al. found a higher sensitivity using CSV (86%) compared to DTV (75%) for detecting 2 mm protrusions [6]. The lower sensitivity of CSV for the detection of 1–2 mm protrusions in the current study might be due to the standard use of DTV at our institution, while CSV is not routinely performed. Bergsma et al. described a learning curve for the acquisition of DTV, which might also be applicable to CSV [39]. However, all protrusions ≥ 2 mm were detected with both tangential views in the current study.

### 4.2. Detection of Intra-Articular Screws in 2D Fluoroscopy

Detection of intra-articular screw misplacement also remains limited in standard fluoroscopic views due to the biconcave articular surface of the distal radius [40].

Takemoto reported a sensitivity of 84.5% using plain radiographs with standard/tilted AP/lateral and 45° pronation views, comparable to the sensitivity of 83.33% found for the combination of tilted AP/lateral and oblique fluoroscopy views in the current study [41]. Tweet et al. observed higher sensitivities for the combination of tilted AP/lateral views (92%) [42]. For single views, the sensitivities for standard/tilted AP views were lower in the current study, while similar sensitivities for standard/tilted lateral views were found [42].

### 4.3. Detection of Screw Misplacement in 3D Fluoroscopy

Even with the use of additional fluoroscopic views, screw misplacement can still be missed [20]. Clinical studies have shown that intra-operative 3D fluoroscopy led to the detection and revision of misplaced screws in up to 31% of cases [18,19,43]. The results of the current study align with previous studies demonstrating a higher sensitivity of 3D fluoroscopy in the detection of screw misplacement.

Wung et al. reported a sensitivity of 81.8% for 3D fluoroscopy in detecting dorsal screw protrusion compared to 72.2% for DTV [44]. In contrast, Langerhuizen et al. found a lower sensitivity for 3D fluoroscopy compared to conventional fluoroscopy, including DTV, although a reduction in dorsally penetrating screws in postoperative CT scans was observed in this study for 3D fluoroscopy compared to 2D fluoroscopy [20]. It has to be noted that patients from different cohorts were included in this study, limiting direct comparison of 2D and 3D fluoroscopy.

In the detection of intra-articular screws, Borggrefe et al. found a higher sensitivity using a 3D C-arm compared to plain radiographs. However, the reported sensitivity of 88% was slightly lower than the 92% observed in the current study [45]. In contrast, Seuthe et al. reported a sensitivity of 100% for both 3D and 2D fluoroscopy (AP/lateral) in a cadaveric model [46].

For dorsal screw protrusions, a reduction in the missed screw rate from up to 37% with 2D fluoroscopy to 18.2% with 3D fluoroscopy may help to reduce the risk of EPL tendon irritation and rupture in clinical practice [8,44].

Previous studies have also demonstrated that unicortical fixation of distal locking screws provides sufficient stability in volar locking plates and may reduce the risk of dorsal screw protrusion. Several authors have therefore recommended using screws that are 2 mm shorter than the measured depth to minimize the risk of protrusion [25,34,47].

### 4.4. Radiation Exposure

Although the DAP was significantly higher with 3D fluoroscopy, it should be noted that the OR personnel leave the controlled area during scan acquisition, minimizing occupational radiation exposure. In contrast, the fluoroscopy time, radiation dose, and accuracy for conventional fluoroscopy depend on the surgeon’s experience. The DAP for conventional fluoroscopy in this study was similar to the radiation exposure reported in clinical settings, although foam positioning aids were used in the current study to facilitate the acquisition of tilted views [19]. Overall, while 3D fluoroscopy increases intraoperative radiation exposure compared to conventional 2D imaging, previous studies suggest that it might lead to a decrease in postoperative CT scans [48].

Some studies have investigated ultrasound as a potential alternative to detect dorsal screw protrusion without radiation exposure [28]. However, reports on sensitivity and inter-rater reliability remain inconsistent, and additional fluoroscopy is still required to assess implant positioning and fracture reduction [30,37].

### 4.5. Limitations

The current study has several limitations. The cadaveric model does not enable an assessment of clinical outcomes, and the small number of specimens limits the transferability of the results. However, based on observed effect sizes, the sample size provided sufficient statistical power (>0.88) to detect differences in the detection of dorsal screw protrusion when using 2D and 3D fluoroscopy, while power was lower for intra-articular screw detection (0.66) due to smaller effect sizes.

Since dorsal protrusion was induced simultaneously in two screws in each specimen, an analysis of location-specific detection rates was not possible. Future studies exchanging single screws are needed to evaluate detection differences, particularly in the third extensor compartment. Additionally, the sensitivity of oblique views should be evaluated separately for each screw location, as supinated and pronated views are typically used to facilitate the detection of screw protrusion into the second and fourth compartments, respectively.

The high detection rate of intra-articular screws using 2D fluoroscopy in our study may be due to the average protrusion of 2 mm and the exchange of a single screw, which significantly altered the screw trajectory, raising suspicion about a possible screw penetration. Furthermore, the use of intact radii, as opposed to fractured specimens, may have facilitated the detection of screw misplacement. Further studies using fractured distal radius specimens are warranted to more realistically assess screw detection performance under clinical conditions.

Interestingly, in some cases, the sensitivity for detecting dorsal screw protrusion was higher with single views (supination or DTV) than with the combination of several images, despite using the same images. It is possible that the observers were distracted by the other views and therefore did not identify the dorsal screw protrusion consistently.

Although 3D fluoroscopy may lead to an improved detection of misplaced screws, it has to be noted that it may not be readily available in every hospital.

## 5. Conclusions

Three-dimensional fluoroscopy provides a higher sensitivity for detecting intra-articular and dorsal screw protrusion at the distal radius compared to 2D fluoroscopy. However, despite its advantages in the detection of screw misplacements, it has limitations, including increased radiation exposure and limited availability, which restrict its use to selected cases. In cases in which 3D fluoroscopy is not performed, the use of DTV can improve the detection of dorsal screw protrusion, and the use of oblique views can enhance the detection of intra-articular screw penetration. Further research is needed to establish a standardized intraoperative imaging protocol for distal radius fractures.

## Figures and Tables

**Table 1:** Likert scale for rating of observer confidence.

Rating	Observer Confidence
1	Very low
2	Low
3	Moderate
4	High
5	Very high

**Table 2:** Detection of dorsal screw protrusion in 3D fluoroscopy and combinations of different conventional fluoroscopic views. Observer confidence is shown as median (IQR).

	3D (*n* = 18)	Set 1 (AP/Lat/Pro/Sup) (*n* = 18)	Set 2 (AP/Lat/CSV/DTV) (*n* = 18)	Set 3 (AP/Lat/Pro/Sup/CSV/DTV) (*n* = 18)
**Sensitivity (%)**				
overall	97.22	63.89	75.00	77.78
1–2 mm	94.44	38.89	50.00	55.56
> 2 mm	100	88.89	100	100
**Specificity (%)**	100	77.78	88.89	83.33
**Fleiss’ Kappa**	0.92	0.63	0.70	0.62
**Observer confidence**	5 (5–5)	4 (2–5)	4 (3–5)	5 (4–5)

**Table 3:** Comparison of single 2D fluoroscopy views for the detection of dorsal screw protrusion. Observer confidence is shown as median (IQR).

	Lateral (*n* = 18)	Lateral Tilt (*n* = 18)	Supination (*n* = 18)	Pronation (*n* = 18)	DTV (*n* = 18)	CSV (*n* = 18)
**Sensitivity (%)**						
overall	58.33	52.78	97.22	33.33	100	69.44
1–2 mm	50.00	22.22	94.44	33.33	100	38.89
>2 mm	66.67	83.33	100	33.33	100	100
**Specificity (%)**	66.67	83.33	61.11	83.33	94.44	83.33
**Fleiss’ Kappa**	0.33	0.62	0.68	0.08	0.91	0.93
**Observer confidence**	4 (3–5)	4 (2.75–4)	5 (3–5)	3 (2–4)	5 (4–5)	5 (4–5)

**Table 4:** Detection of intra-articular screw penetration. Observer confidence is shown as median (IQR).

	3D (*n* = 22)	Set 1 (AP/Lat) (*n* = 19)	Set 2 (AP/Lat/Pro/Sup) (*n* = 19)
**Sensitivity (%)**	95.83	79.17	83.33
**Specificity (%)**	96.97	87.88	93.94
**Fleiss’ Kappa**	0.86	0.71	0.85
**Observer confidence**	5 (5–5)	4 (3–5)	4 (3–5)

**Table 5:** Comparison of 2D fluoroscopic views for the detection of intra-articular screw penetration. Observer confidence is shown as median (IQR).

	AP (*n* = 11)	AP Tilt (*n* = 11)	Lateral (*n* = 11)	Lateral Tilt (*n* = 11)	Supination (*n* = 11)	Pronation (*n* = 11)
**Sensitivity (%)**	57.58	75.76	60.61	69.70	72.73	84.85
**Specificity (%)**	72.73	78.79	72.73	96.97	93.94	100
**Fleiss’ Kappa**	0.87	0.58	0.58	0.33	0.75	0.69
**Observer confidence**	3 (2–4)	3 (2–5)	2 (1–3)	2 (1–3)	3 (2–4)	3 (2–4)

## Data Availability

All data and statistics are available upon request from the corresponding author.

## Abbreviations

The following abbreviations are used in this manuscript:

AP	Anteroposterior.
CBCT	Cone beam computed tomography.
CSV	Carpal shoot through view.
DAP	Dose area product.
DTV	Dorsal tangential view.
EPL	Extensor pollicis longus.
ICC	Intraclass correlation coefficient.
IQR	Interquartile range.
